# Endoscopic Surgery under Fluoroscopic Guidance Is Useful for Diagnosing and Treating Epiphyseal Osteomyelitis Caused by *Mycobacterium* Species

**DOI:** 10.1155/2018/8136150

**Published:** 2018-06-13

**Authors:** Hironori Ochi, Katsuaki Taira, Naho Nemoto, Noboru Oikawa, Soya Nagao, Tadamasa Takano, Kazuo Kaneko

**Affiliations:** ^1^Department of Orthopaedic Surgery, Saitama Children's Medical Center, Saitama, Japan; ^2^Department of Orthopaedic Surgery, Juntendo University School of Medicine, Tokyo, Japan; ^3^Department of Orthopaedic Surgery, Nihon University School of Medicine, Tokyo, Japan; ^4^Department of Infectious Diseases and Immunology, Saitama Children's Medical Center, Saitama, Japan

## Abstract

Osteomyelitis caused by *Mycobacterium* species may be difficult to diagnose and treat. We report a case of treatment for osteomyelitis caused by *Mycobacterium* species in the epiphysis of the right proximal tibia. A 28-month-old boy presented to a hospital with symptoms of fever and right knee pain. He had been vaccinated with *Mycobacterium bovis* Bacille Calmette-Guérin (BCG) at five months of age. The epiphyseal radiolucent lesion had increased in size and extended to the metaphysis through the physis on a plain radiograph of the right proximal tibia. Surgical drainage and curettage of the lesion were performed with an endoscope under C-arm fluoroscopy. The intraoperative histopathological examination revealed granulation tissue composed of caseous necrosis and Langerhans giant cells, revealing *Mycobacterium* species to be the causative pathogen. Because of suspected osteomyelitis caused by BCG, the antituberculosis drugs were administered orally from an early postoperative stage. A plain radiograph taken eight months postoperatively showed bone regeneration in the area of curettage and a slight physeal bridge, in addition to normalization of the inflammatory response on blood sampling. It was possible to perform accurate diagnosis and rapid treatment for epiphyseal osteomyelitis caused by *Mycobacterium* species using endoscopic surgery under fluoroscopic guidance.

## 1. Introduction

Primary epiphyseal osteomyelitis caused by mycobacteria is extremely rare [1]. *Mycobacterium* species should be considered as a possible causative pathogen of primary epiphyseal osteomyelitis, especially when infants or toddlers present with a history of *Mycobacterium bovis* Bacille Calmette-Guérin (BCG) vaccination or pulmonary tuberculosis, and when symptoms are unresponsive to treatment with antibiotics [1]. Although BCG osteomyelitis is a rare complication of BCG vaccination in immunocompetent hosts, it also occurs in healthy toddlers [1, 2]. Osteomyelitis caused by *Mycobacterium* species may be difficult to diagnose and treat [1]. The causes of difficulty in treatment are the lesion being confined to the epiphysis, which is poorly vascularized, much slower response to chemotherapy, and inadequate surgical removal of the lesion [1]. Osteomyelitis is likely to recur for these reasons, and reoperation is often required [1–3]. It is very important and challenging to remove the lesion sufficiently to prevent recurrence. We report a case of treatment for osteomyelitis caused by *Mycobacterium* species in the epiphysis of the right proximal tibia using an endoscopic technique under fluoroscopic guidance.

## 2. Case Report

**Figure 1:**
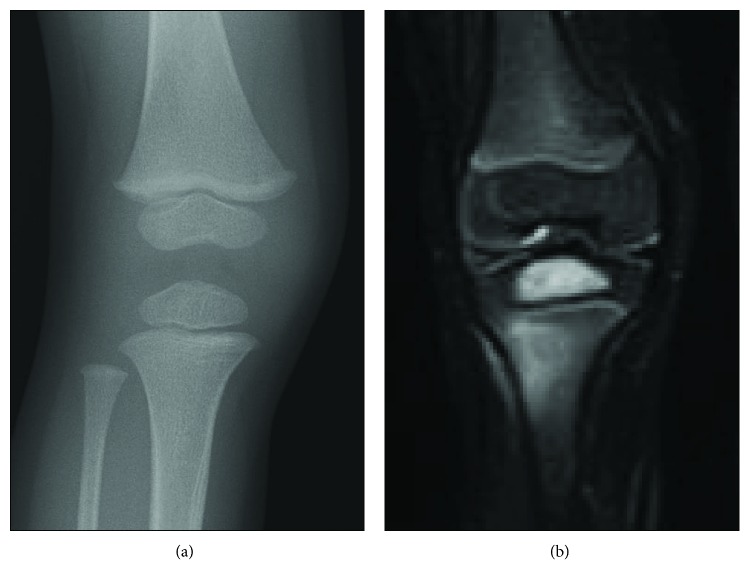
Initial examination. (a) Plain radiograph of the knee showing no apparent abnormalities. (b) MRI demonstrating a high-signal intensity area at the epiphysis of the right proximal tibia in the short tau inversion recovery sequence.

**Figure 2:**
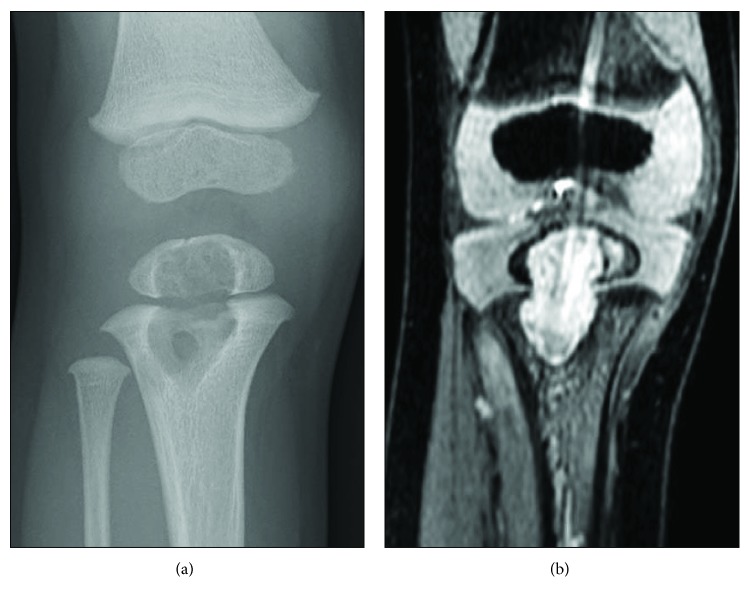
After treatment with intravenous antibiotic chemotherapy. (a) Plain radiograph showing the epiphyseal radiolucent lesion extending to the metaphysis through the physis at the right proximal tibia. (b) MRI demonstrating an abnormality at the same site, extending across the high-signal intensity area of the physis in the three-dimensional fast field echo sequence.

Informed consent was obtained from the patient's parents, and the study was approved by the ethics committee of the Saitama Children's Medical Center. A 28-month-old boy presented to a hospital with symptoms of fever and right knee pain. He had been vaccinated with BCG at five months of age. The patient had no previous history of trauma or illness and no known history of tuberculosis contact. At the time of the initial examination, radiography of the knee showed no apparent abnormalities (Figure 1(a)). Magnetic resonance imaging (MRI) demonstrated a high-signal intensity area at the epiphysis of the right proximal tibia in the short tau inversion recovery sequence (Figure 1(b)). The peripheral white blood cell count (WBC) was 11,100/*μ*L and the serum C-reactive protein (CRP) level was 5.8 mg/dL, indicating a mild inflammatory condition. The blood culture test was negative. After treatment with intravenous antibiotic chemotherapy (cefazolin 100 mg/kg/day) for four weeks, suspecting osteomyelitis, the symptoms of fever and knee pain improved and the WBC normalized. However, the symptom of limping persisted and CRP was slightly high (CRP 0.34 mg/dL). The patient was referred to our hospital two months after initial presentation of symptoms. The epiphyseal radiolucent lesion had increased in size and extended to the metaphysis through the physis on a plain radiograph of the right proximal tibia (Figure 2(a)). MRI of the knee demonstrated an abnormality at the same site, extending across the high-signal intensity area of the physis in the three-dimensional fast field echo sequence at the right proximal tibia (Figure 2(b)). Surgical drainage and curettage of the lesion was carried out in order to confirm the diagnosis and remove the lesion at five months after initial symptoms.

## 3. Surgical Technique

**Figure 3:**
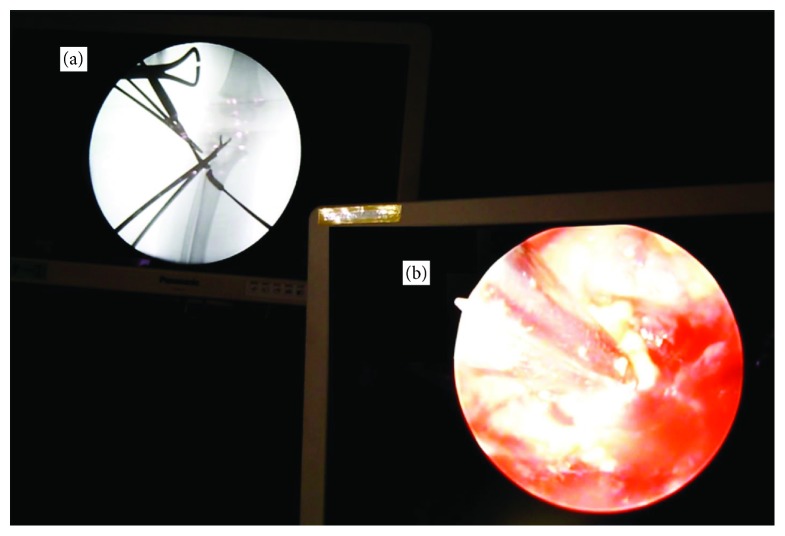
Surgical drainage and curettage of the lesion were performed with an endoscope under C-arm fluoroscopy using double monitors. (a) Fluoroscopic monitor. (b) Endoscopic monitor.

**Figure 4:**
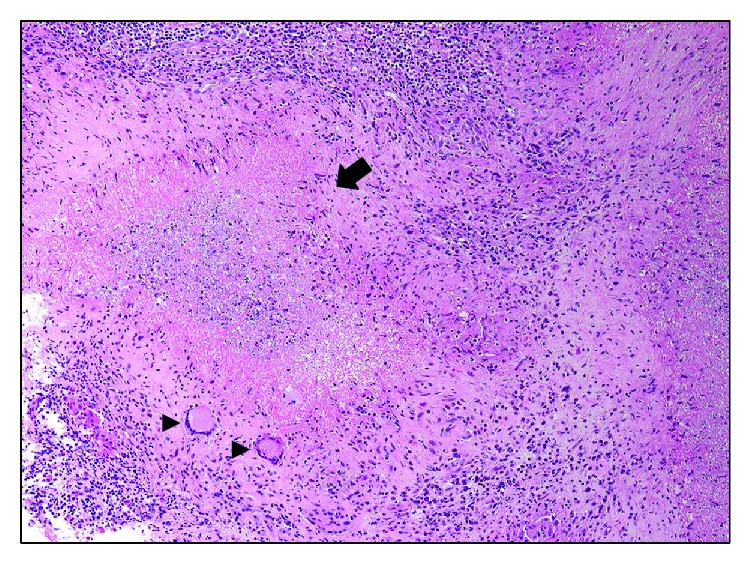
Intraoperative histopathological examination (hematoxylin-eosin stain, ×4). Arrow: caseous necrosis. Arrowhead: Langerhans giant cells.

**Figure 5:**
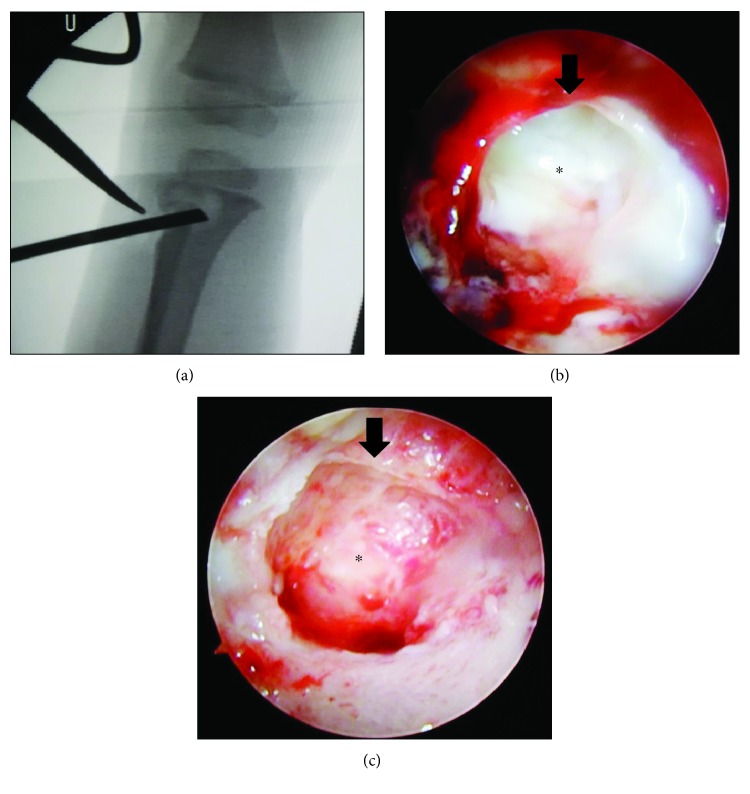
Endoscopic view from metaphysis to epiphysis through physis. (a) Endoscopic view under fluoroscopic guidance. (b) Lesion before drainage. (c) Lesion after drainage. Arrow: physis. ^∗^Epiphysis.

Surgical drainage and curettage of the lesion were performed with an endoscope under C-arm fluoroscopy (Figure 3). A 2 cm skin incision was made under fluoroscopic guidance at the lateral aspect of the proximal tibial metaphysis. A bone tunnel was created and a 4.0 mm-diameter endoscope was inserted. A punch was inserted into the same tunnel to remove a tissue sample for intraoperative histopathological examination. The lesion contained granulation tissue composed of caseous necrosis and Langerhans giant cells (Figure 4). Ziehl-Neelsen staining failed to identify any acid-fast bacteria. Curettage was advanced in the direction of the physis. The bone defect within the growth plate was identified by palpation with a probe. The debridement was performed especially gently around the growth plate. The endoscope was inserted deeper through the bone defect of the physis, and the inside of the epiphysis was examined. The abnormal tissue inside the epiphysis was carefully debrided. After confirming adequate removal of the lesion, the surgery was finished (Figure 5).

## 4. Postoperative Treatment

**Figure 6:**
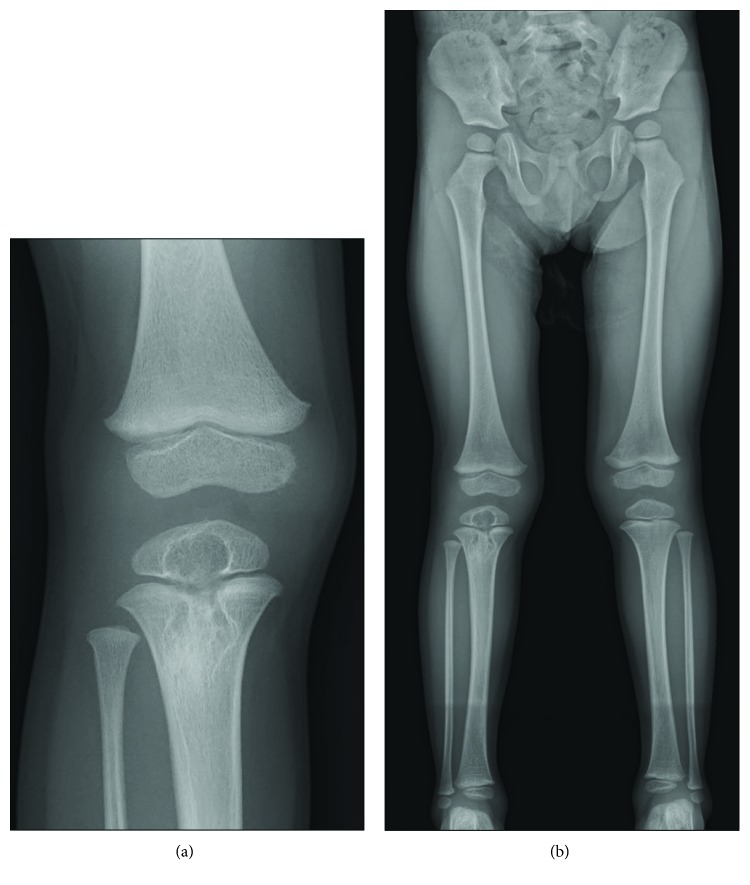
Eight months postoperatively. (a) Plain radiograph showing bone regeneration in the area of curettage and a slight physeal bridge at the right proximal tibia. (b) Growth disturbance and deformation were not seen in a full-length lower-limb radiograph.

Because of suspected osteomyelitis caused by BCG, the antituberculosis drugs (isoniazid 10 mg/kg/day and rifampicin 15 mg/kg/day) were administered orally from an early postoperative stage. The symptoms of limping disappeared after surgery. Mycobacterial cultures could not identify *Mycobacterium* species and polymerase chain reaction (PCR) could not be performed. A plain radiograph taken eight months postoperatively showed bone regeneration in the area of curettage and a slight physeal bridge, in addition to normalization of the inflammatory response on blood sampling with the administration of antituberculosis drugs (Figure 6(a)). Growth disturbance and deformation were not seen in a full-length lower-limb radiograph (Figure 6(b)).

## 5. Discussion

Primary epiphyseal osteomyelitis caused by mycobacteria is extremely rare [1]. Furthermore, the diagnosis and treatment are difficult because osteomyelitis related to *Mycobacterium* species often results in mild or no systemic manifestations, inconclusive laboratory studies, and culture-negative bone specimens [4]. In the current case, it was possible to accurately diagnose and rapidly treat epiphyseal osteomyelitis using endoscopy from the results of an intraoperative histopathological examination. It is very important to administrate antituberculosis drugs from an early postoperative stage without waiting until the results of culture or PCR are known. However, although the recurrence of osteomyelitis could be prevented in this case, it might be difficult to avoid complications such as physeal damage caused by osteomyelitis.

A tissue sample was collected from the lesion by endoscopy, and the intraoperative histopathological examination revealed granulation tissue composed of caseous necrosis and Langerhans giant cells, revealing *Mycobacterium* species to be the causative pathogen. It could then be judged that the curettage was performed sufficiently and that early postoperative antituberculosis medication was necessary. Since the patient with a history of BCG vaccination had no history of tuberculosis contact and was not living in high tuberculosis burden countries [5, 6], BCG osteomyelitis was primarily suspected. As causes of epiphyseal osteomyelitis other than *Mycobacterium* species, *Kingella kingae*, a Gram-negative bacterium, is the leading cause of bone and joint infections in early childhood [4, 7]. *Kingella kingae* has an unexplained affinity for the knee and epiphysis of long bones [4, 7]. It is also essential to exclude tumors by image or histopathological studies [4]. Mycobacterial cultures and PCR are sometimes inadequate to prove a diagnosis postoperatively [3, 4, 8]. In contrast, intraoperative histopathological examination of bone lesions is effective for diagnosis and allows rapid treatment. For this purpose, it is necessary to collect sufficient quantities of appropriate specimens, and endoscopic surgery is very useful for this as it offers a direct view. Since delaying the administration of antituberculosis drugs may increase the risk of recurrence of osteomyelitis caused by *Mycobacterium* [2], it is important to diagnose and to administer antituberculosis drugs from the earliest possible postoperative stage.

In the current patient, we could perform the debridement sufficiently and gently for epiphyseal osteomyelitis using an endoscope with a direct view. Yoo et al. reported performing debridement using only fluoroscopy [1]. However, that method may remove the lesion inadequately and damage normal tissues such as the cartilage and growth plate. Saisu et al. reported that debridement for epiphyseal osteomyelitis was performed effectively using an endoscope with two portals [9]. In the current case, we used only one portal, reducing the invasiveness, and we could operate efficiently by inserting the endoscope and punch via the same bone tunnel (Figure 3). The epiphyseal osteomyelitis caused by *Mycobacterium* had extended to the metaphysis through the physis, so although the recurrence of osteomyelitis could be prevented by endoscopic gentle debridement, it was difficult to avoid physeal damage. It is important to carefully monitor growth disturbances and deformation and to perform additional surgery such as physeal bridge resection or corrective osteotomy, depending on each situation [1, 8].

A limitation to this study was that the follow-up period was short. However, some previous reports describing the period from the first surgery to the recurrence of osteomyelitis noted that most recurrences of osteomyelitis occurred within six months [2, 3, 9]. Thus, the possibility of recurrence of osteomyelitis was considered to be low in this case. Although growth disturbances and deformation require long-term follow-up, it is important to recognize the possibility of these complications and how to treat them at the time of initial treatment.

## 6. Conclusion

It was possible to perform accurate diagnosis and rapid treatment for epiphyseal osteomyelitis caused by *Mycobacterium* species using endoscopic surgery under fluoroscopic guidance. It is important to recognize the specific complications such as growth disturbances and deformation that may occur due to this epiphyseal osteomyelitis.
